# Educating for interprofessional practice: moving from *knowing* to *being*, is it the final piece of the puzzle?

**DOI:** 10.1186/s12909-016-0844-5

**Published:** 2017-01-06

**Authors:** Helena Ward, Lyn Gum, Stacie Attrill, Donald Bramwell, Iris Lindemann, Sharon Lawn, Linda Sweet

**Affiliations:** 1 School of Medicine, The University of Adelaide, Adelaide, South Australia Australia; 2 School of Medicine, Flinders University, Adelaide, South Australia Australia; 3 Speech Pathology, School of Health Science, Flinders University, Adelaide, South Australia Australia; 4 Flinders International Study Centre, Flinders University, Adelaide, South Australia Australia; 5 Health Professional Education, School of Medicine, Flinders University, Adelaide, South Australia Australia; 6 School of Nursing and Midwifery, Flinders University, Adelaide, South Australia Australia

## Abstract

**Background:**

Professional socialisation and identity arise from interactions occurring within university-based interprofessional education, and workplace-based interprofessional practice experience. However, it is unclear how closely language and concepts of academic learning situations align with workplace contexts for interprofessional learning. This paper reports on a study that brought together university-based educators responsible for teaching health professional students and health service-based practitioners who supervise students in the field.

**Methods:**

Interviews and focus groups with university-based educators and health service-base practitioners were used to explore perceptions of capabilities required for interprofessional practice. The qualitative data were then examined to explore similarities and differences in the language used by these groups.

**Results:**

This analysis identified that there were language differences between the university-based educators and health service based practitioners involved in the project. The former demonstrated a curriculum lens, focusing on educational activities, student support and supervision. Conversely, health service-based practitioners presented a client-centred lens, with a focus on communication, professional disposition, attitude towards clients and co-workers, and authenticity of practice.

**Conclusions:**

Building on these insights, we theorise about the need for students to develop the *self* in order to *be* an interprofessional practitioner. The implications for health professional education in both university and workplace settings are explored.

## Background

Interprofessional Practice (IPP) occurs when all members of the health service delivery team participate in the team's activities and rely on one another to accomplish common goals and improve health care delivery, improving the quality of the patient's experience. Interprofessional practice is the result when interprofessional learning is put into practice in the health workplace and in the community [1]. Interprofessional Education (IPE) has been defined as occuring when “two or more professions learn with, from and about each other to improve collaboration and the quality of care" [2].

Delivering the education required to foster capabilities for IPP has been the subject of substantial publication, discussion and development in recent years. Ways of promoting and assessing IPP have received increasing focus within health professions courses, professional associations, governments and others charged with developing and implementing health policy, and workforce development [3–5].

University programs, particularly in health professional courses, seek to ensure relevance to practice and students’ preparedness for the workplace by including a balance of university and workplace-based elements. These elements perform different learning functions that enable the development of conceptual and procedural knowledge, and facilitate socialisation and the construction of professional capabilities [6, 7]. The conceptual prominence and values emphasised within university learning environments are different to those of workplace learning environments, where service delivery and client needs have primacy over the needs of the learner. However, these different purposes, concepts and values, and the nature of the learning that occurs within these different situations, may not be transparent for the student [8, 9].

Students’ participation in the workplace provides exposure to professional attitudes and behaviours, and promotes the development of practical knowledge and skills [10]. Workplace experiences should provide learning that complements university-based teaching and should be developed, structured and evaluated for the contribution they make to students’ development [7, 11]. Learning in the workplace can include intentional and unintentional learning [7, 11, 12]. Workplace learning can address formal curricular outcomes as well as informal, or hidden learning, and expose students to implicit values and assumptions held by practitioners [13]. Through the process of socialisation within the workplace, newly acquired values, attitudes and behaviours may not necessarily be congruent with those of the university [14].

While intentional learning can be covered by a formal curriculum, unintentional learning has been characterised as either informal or hidden [15]. Informal curriculum is interpersonal, occurring in interactions between students and teachers (both practitioners and academics) not directly related to the curriculum, and the hidden curriculum is ‘a set of influences that function at the level of organizational structure and culture’ ([15], p. 404). Informal learning can include implicit assumptions and expectations of others which are transmitted though behaviours and conversations as relationships are developed in the workplace [16]. It is possible that informal learning, which might have the greater impact on the development of attitudes towards, and assumptions about patients and other professionals, is underemphasised in university courses. It is unclear to what extent the informal learning which occurs through the use of language about IPP is complementary or discordant across university-based and practice-based settings, and whether this is important for student learning. It is also unclear how the use of language about IPP is reflected in the hidden curriculum which is dependent on the culture within an organisation [15] and the basic assumptions about practice and learning shared within an organisation ([17], p.18).

Gum et al. [18] set out to bring together university-based educators (referred to as educators), who are responsible for teaching health professional students in universities, with health service-based practitioners (referred to as practitioners), who are responsible for supervising students in the workplace, to explore their perceptions of interprofessional capabilities [18]. In a previous publication Gum at al presented an interprofessional capabilities framework, recommending three core capabilities that students need to develop to enable them to be interprofessional practitioners; namely, client focused care, collaborative skills and awareness of own and other professions [18]. These three primary capabilities were depicted as enveloped by the need for respect, values and communication. At the time of developing the framework, the connection between respect, values and communication and the three core capabilities could not be described in detail from the original analysis, although there was evidence that all are necessary in developing students for interprofessional practice.

During the data analysis for the primary study and framework development, the researchers noticed that the language used by educators to describe interprofessional capabilities differed to that used by practitioners. This prompted further exploration of the data to attend to the form of language used by these two groups, as both are key to enacting successful interprofessional education for students. Whilst the primary study utilised action research methodology to discover, construct and reflect about educators’ and practitioners’ understanding of interprofessional capabilities, this subsequent analysis explored the data through a different lens, attending specifically to the language used by educators and practitioners. Therefore, this subsequent analysis draws on the same data corpus, to address the research question:

- What language do educators and practitioners use when describing interprofessional capabilities?

This paper will present and discuss the findings related to language use and explore their relevance for health professional education in both university and practice settings.

## Methods

The primary study adopted an action research methodology using focus group discussions and semi-structured interviews with educators and practitioners to explore their perspectives about IPP and is reported by Gum, et al. [16, 18]. The primary study aimed to clarify the capabilities students need to learn through their academic and workplace-based learning, supporting curriculum development. In the secondary analysis of the data, we applied discretely different methods from the primary analysis. Described as a ‘sequential analysis’, we explored different meanings in the data related to the language used by educators and practitioners to describe IPP [19].

The primary study grew from a partnership between a primary health network and a university, and was situated within a new community-based healthcare service in South Australia, which was mandated to embed interprofessional practice principles within service delivery. A reference group was formed to support the project, represented by members from the university and healthcare service. The reference group constructed the project and spent shared time to identify its purpose, reflecting a desire to improve the “space” between the needs of the university and practitioners in the field to support students to develop IPP skills. An action research framework, using a participatory and collaborative approach was chosen as the research method [20]. This supported the reflective vision espoused by the reference group, enabling the perspectives of educators and practitioners to unfold through iterative and active cycles of planning, acting, reflecting and observing. The reference group appointed a project manager who was intentionally embedded and habituated within both the healthcare setting and the university. This supported the development of relationships and ongoing engagement of stakeholders, providing feedback between the settings and the reference group, and informing the reflective process.

The research team was comprised of members of the reference group (HW, LG, IL, SL) and members not involved in the reference group (SA, DB, LS). This enabled the perspectives of project ‘insiders’ through the reference group, as well as the distinct and critical viewpoint taken by members more distant from the project. These close and distant perspectives supported critical reflection about the research process, and enabled verification and interpretation of findings identified through thematic analysis.

### Participants

The members of the reference group were involved in identifying participants and facilitating interviews, but were not interview participants. Interview participants were purposively sampled including clinicians from the healthcare facility; and academic staff involved in teaching health professional students at the university, representing a wide variety of disciplines as shown in Table 1.

**Table 1:** Participant disciplines

Educators	Practitioners
Disability and community inclusion	Health promotion
Health professional education	Nursing (clinical, health promotion & management)
Health promotion	Nutrition & Dietetics
Medicine	Oral health
Midwifery	Paramedics
Nursing	Podiatry
Nutrition & dietetics	Clinical psychology
Optometry	Social work
Paramedicine	Speech pathology
Speech pathology	Youth workers
Surgery	
Psychology	
Social work	

As the action research aimed to support shared understanding and community ownership of IPP within the new healthcare setting, broad invitations were distributed. Participants attended voluntarily and were not always those involved in clinical teaching with students. Similarly, participants attended from a variety of discipline backgrounds, with new and previous participants invited for each cycle, enabling breadth of perspective. This also supported extensive dissemination of the knowledge constructed about IPP to stakeholders. Table 2 summarises the distribution of practitioners and educators in the interviews across the cycles.

**Table 2:** Summary of focus groups and interviews

Cycle	Date	Activity	Practitioners	Educators
1	August – September	Focus groups 1, 2	11	0
	2011	Focus groups 3–5	0	14
2	October – December	Focus group 1–3	20	0
	2011	Focus group 4, 5	0	11
		Interviews	1	6
3	April 2012	Focus group 1	11	0
		Focus group 2	10	4

### Cycles of data collection

Data were collected through focus group discussions (one hour duration) and semi-structured interviews (20 – 30 min duration) conducted over three iterative cycles. These encouraged joint ownership of project outcomes and allowed the research team to build on learning over time and to develop and enhance academic-workplace partnerships through ongoing engagement. Discussions were facilitated by two moderators who were members of the reference group (HW, IL). The action research cycles and detailed discussion of the methods used in this project are reported elsewhere [18]. In summary, the first cycle explored the educators’ and practitioners’ perceptions of IPP separately through a series of focus groups and interviews. The second cycle enabled opportunity to reflect about, and expand on the developing understanding through presentation of the findings and the capability framework. The final cycle provided an opportunity to interview educators and practitioners together in order to achieve collaboration and develop consensus. A framework of interprofessional capabilities evolved from the findings (reported in Gum et al.) [18]. Interviews identified strategies and explored implementation of the framework within education and healthcare settings. Participants were also invited to explore the themes identified with examples from their own experiences, and to provide alternative viewpoints which were integrated into the findings.

### Data analysis

The primary study used action research cycles to iteratively construct, reflect on and consolidate shared meaning across the stakeholders embedded within the project [18]. These early findings provided the formative context for a ‘sequential analysis’ or second round of analysis on the same dataset [19]. This utilised Gale and colleagues’ [21] framework analysis method to explore the language of the educators and practitioners about IPP. This assisted us to understand what and how ideas about interprofessional practice were expressed, as opposed to the knowledge construction aims of the primary action research study. Conducting the analyses sequentially enabled us to attend to knowledge construction and language use in a way that was complementary. This allowed us to view two different aspects of IPP, bringing into view discrete perspectives of the phenomenon.

The framework method was applied to thematically explore the dataset. This is appropriate to identify themes from qualitative data in multidisciplinary health research [21]. All interview data were transcribed, and coding and analysis was completed by three members of the research team (HW, IL and LG). Open coding was used initially to describe the transcribed data, focussing particularly on the language used by educators and practitioners to describe IPP. The codes generated and analysis were then scrutinised by the wider research team, and a coding framework was developed and applied to each transcript. The transcripts were then coded independently, and these were discussed collectively until consensus was achieved. Dialogue and discussion within the research team supported a constant comparative process of codes and themes identified, ensuring that these were grounded in the data. The team then convened and collaborated to interpret themes from the data, placing these within the context of the interprofessional capabilities identified in the initial study. We were therefore able to build a picture of IPP through exploring the capabilities required as well as the language used to describe those capabilities.

## Results

This analysis focused on the language used by participants and showed that there were differences between the educators and practitioners in their expression of IPP, which were consistent across all the data. On a surface level, there were differences in individual word use. However, it was identified that the language for the two professional groups was embedded in different perspectives of practice. The educators articulated practice using a curriculum lens, with a focus on educational activities, student support and supervision. The practitioners presented practice through a client-centred lens, with a focus on communication, professional disposition, attitude towards clients and co-workers, and authenticity of practice. The findings will be presented under the following headings: *language use*; *educators’focus for IPP*; and *practitioners’ focus for IPP* using quotes representative of the views of each group. This will be followed by a discussion of how these findings and the literature build on our understandings of educating for interprofessional practice.

### Discussion of Identity

The educators were articulate in discussing the knowledge, skills and attitudes professionals and students need to possess in order to work well interprofessionally. They spoke of ‘*clear’* and ‘*open communication’* which was ‘*respectful’, ‘timely’* and ‘*employed reflective listening’,* as well as being ‘*aware’* of the work of other professional groups, and being ‘*secure’* and ‘*accountable’* in their own profession. Positive ‘*collaborative skills’* and ‘*supportive team behaviours’* were discussed in order to best meet the common goal, which was identified as the needs of the client or community to which the health professional(s) was responsible. When words such as ‘*trust’* and ‘*honesty’* were mentioned, it was generally applied to the professional relationship developed in meeting the needs of the client or community. There was a strong recognition within this group that interprofessional practice was in the best interests of clients and the community, and provided more ‘*efficient, effective’* and ‘*safer care’.* These views concur with the professional literature on interprofessional practice [22–24]. Educators spoke often of the needs of clients as being the ‘*common goal’* which brought practitioners together, but they did not articulate any requirement for building knowledge or developing a relationship on a personal level with other professionals in order to work together ‘*toward a common goal*’.

Some educators expressed the view that each profession has its own distinct language, and that IPE would be problematic because of the different ‘*language’* each profession used. One educator stated:

*If I’m in one speciality, one profession and I’m now going to be hosting, talking to another profession, …, one of the first questions is ‘well who’s going to teach me to speak nurse so that I can actually be credible to these students that I’m supposed to be teaching’.* (Focus Group 7 Nov)

Educators also commented about the emotional impact of interprofessional practice on students, and the importance of understanding what it means ‘*to be’* a practitioner, although what it means ‘*to be’* was not elaborated on. One educator explained:

*That they [students] feel for the first time that, ‘I really understand what it means to be a kind of practitioner’. So I think it’s also quite an affirming experience as well.* (Interview 21 Dec)

Discussions in the workshops with practitioners commenced from a different perspective. When asked what was required to work well interprofessionally, an initial response was, ‘*It depends on what kind of person you are’*. Phrases and words such as ‘*values-based’*, *‘open-mindedness’, ‘being friendly’, ‘authentic’* and *‘solid’* were used frequently. The practitioners placed an emphasis on their preference to know other practitioners ‘*personally’* and develop a level of ‘*trust’* with them so that if they were to refer clients to them, they would feel confident that the other professional would be the ‘*right*’ person to meet the needs of their client. This view is in keeping with a values-based approach to practice, as discussed by Adshead [25], in which the values of the client, practitioners, and the context of practice are taken into account in order to develop a positive therapeutic relationship.

Practitioners spoke of the importance of ‘*knowing who you are and what you value’* in order to work interprofessionally with others.

*The respect is very important. It should be okay as to what you bring to the team. Everyone’s had different levels of training and different levels of time in the health sector and different levels of working with clients. … I don’t necessarily see striving for equality as a good, useful thing to do with your time. But striving for respect, I think, is really important*. (Focus Group 12 Oct)

Practitioners expressed the importance of building personal and professional relationships within the team. They focused on students acquiring a balance of skills which included both tangible and interpersonal:

*But equally so, they have an equal amount of skill in what’s more non-tangible, the interpersonal, being able to react and interact to who’s in front of them.* (Focus Group 12 Oct)

They stressed that informal encounters such as ‘*corridor chats’* were important to develop an awareness of each other and a sense of belonging. Some practitioners noted that they felt that the benefits of these informal encounters were not always recognised.

As with the educators, some of the practitioners viewed each profession as having their own language, as illustrated by the following quote:

*But there is sometimes gaining that awareness of willingness to learn other terminologies that different professions use, their lingo, and that checking for understanding is really important, because what means something in one discipline doesn’t necessarily translate to meaning the same thing in others.* (Focus Group 12 Oct)

### Educators’ focus for IPP

There was a curriculum focus for educators, and they spoke of a *‘demarcation between coursework and placement’.* They also perceived pressure to include interprofessional as well as uniprofessional content in the curriculum. One educator explained:

*Because we’re so curriculum driven I think, on our own discipline curriculum and we don’t tolerate, or we don’t feel we have the time for us, if the content has got too much nursing or other disciplines we feel like our students are missing out, they’ve got to learn.* (Focus Group 7 Nov)

Educators focused on what students were ‘*required to achieve*’ or ‘*do*’ whilst they were on placement, as opposed to emphasising what the students might be thinking or feeling about their practice. There was an expectation of a tangible learning outcome that could be stated and made explicit. One educator said:

*I think that students need to be able to write some objectives around what they’re going to learn, so they need to know what the environment is and what they’re going to do and how it’s going to work. So it seems a reasonable thing for the organisation to say ‘this is who we are, these are the sorts of things that go on here, while you’re here you’ll do one of these and you’ll meet with these people’.* (Interview 19 Dec)

The educators viewed the interprofessional practice agenda as important, but as separate to their discipline-specific objectives. An educator explained:

*I guess we have a strong demarcation between coursework and placement, I think the coursework stuff is us getting our house in order as a faculty around our teaching and curriculum in an interdisciplinary way. With placement I actually think you need to get interdisciplinary teaching on the agenda for practitioners, that’s another kind of area of negotiation for you and your team.* (Focus Group 7 Nov)

The importance of feedback from other professions for assisting students to develop their interprofessional capabilities was discussed, including encouraging the student to reflect on their behaviour and the impact it has on the client.

*I think it would be really useful for students to get feedback just in general context from somebody that’s not their placement educator. And giving them feedback outside of the context of the profession, even if you had no idea if what they were saying was correct or not. General feedback, in terms of have you thought about perhaps the way that you said that, how perhaps a client might feel about that.* (Interview 19 Dec)

### Practitioners’ focus for IPP

The practitioners participating in the interviews and workshops described students on placements as needing to have or display particular values or personal qualities. Participants spoke frequently of concepts about the ‘self’ and how these relate to client care. More specifically, they highlighted personal qualities that are required of the individual to perform IPP.

Personal traits including self-awareness, were discussed as being important for IPP. The following practitioner’s quote shows the value placed on the self:

*… a certain kind of person that is attracted to that respectful, flexible way of working. It would be a tricky thing, but I think very worthwhile to get the concepts of self-awareness, power and control.* (Focus group 16 Nov)

A number of practitioners talked about respect, and having a client-centred approach to practice as being inherent in one’s capacity to be an interprofessional practitioner. The following quote shows how a practitioner spoke of being client-centred as a personal attribute, rather than merely a learnable skill. The practitioner explained:

*Some really important personal characteristics that are way more than just being able to communicate with someone. Client focus, to me, isn’t a skill. That’s like a philosophy you might have.* (Focus Group 12 Oct)

Another participant described the client-centred values required for the kind of community-based work they do:

*My view is that people who work in community in challenging areas do tend to be fairly client-friendly or client-focused, because we work in community and primary healthcare. When you work in a hospital or an acute setting, the other personality in the room is the hospital system and the hospital cultures*. (Focus Group 12 Oct)

One practitioner acknowledged, that having certain values could be in conflict with an organisation if they were not supported:

*I think having a bit of an understanding that it’s okay to sit and have a coffee up here with a worker, and that you’re actually building relationships for this purpose, that that culture is there that it’s actually okay, and that that’s supported.* (Focus Group 17 Nov)

The final concept of the ‘self’ described by some practitioners was authenticity. Authenticity was seen as a critical component for students to develop within their own practice, to enable them to provide interprofessional practice.

*If I think of a therapeutic relationship, authenticity is part of that, and that authenticity, that notion encompasses some of that, some of that questioning.* (Interview 17 Nov)

## Discussion

### Enhancing our understanding

**Fig. 1:**
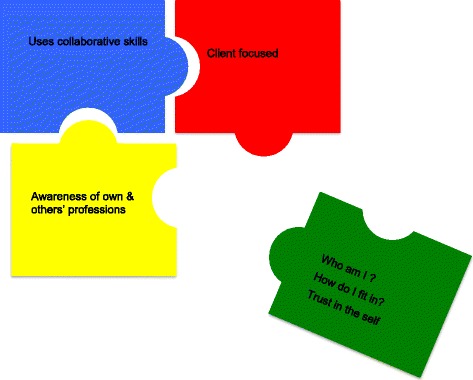
The interprofessional practitioner

The analysis of participant discussions has revealed similarities and differences in the way interprofessional practice is articulated by educators and practitioners. Both groups recognised similar components of interprofessional practice, but used different descriptions to articulate the details and key features of what students should learn. The framework developed to represent the capabilities required for IPP (Gum et al., [18]) (client focused care, collaborative skills and awareness of own and other professions) has been enhanced by these new outcomes [18]. This further analysis of data, has enabled the identification of an additional capability required for IPP which relates to the emphasis by practitioners on the ‘*self’* (see Fig. 1, The interprofessional practitioner).

The practitioner participants clearly spoke of the need for students to develop the *self* in order to *be* an interprofessional practitioner. Practitioner participants expressed that IPP encompasses more than ‘what I know’ rather it is about ‘who I am’ as a practitioner. Within this capability, aspects of the *self,* such as understanding self (who am I?), how one’s self fits within the healthcare team (how do I fit?), and developing trust and confidence in self as practitioner (self-awareness and self-efficacy), are required to enable IPP. If the development of the *self* is a vital IPP capability, the dilemma then is how to theorise about developing the *self* for IPP and how this theory might be integrated into IPE programs.

The different expressions of IPP are not surprising given the different roles of the participants. The identification of these themes do however raise further questions about how this may impact on the preparation of undergraduate health professional students for IPP. These findings will now be explored further through an educational focus with acknowledgement that there may be other ways in which these findings can be interpreted.

The work of Hooper (2014) which emphasises the importance of educators and students developing an awareness of the *self* in interprofessional learning can inform the understanding of these findings [26, 27]. In IPE, students can undergo epistemological transformation, by reflecting on their course content, their experiences in the clinical setting, and their inner selves [26]. Hooper and colleagues [27], p.472] proposed an integrative learning taxonomy for health science education, in which’students connect their inner and outer experiences’. Using this approach, students are encouraged to link elements such as experiences and needs of clients, and professional reasoning with the development of their own professional identity.

Hooper’s scholarship on the epistemological foundations of IPE has been informed by the works of Kegan and Baxter Magolda [28, 29]. Kegan described three dimensions in self-authoring: epistemological change, whereby *how* the learner knows is transformed as distinct from *what* the learner knows (similar to our concepts of *who I am* versus *what I know*); relationships with others, known as the interprofessional; and relationship with the self, known as the *intraprofessional* [29]. An example of how the self-authoring approach can be applied to assist the development of the ‘self’ is within Baxter Magolda’s Learning Partnerships Model, in which learners are supported to develop in the dimensions of their personal epistemology, relationships (interpersonal) and sense of self (intrapersonal) [28]. This pedagogical approach provides a way for students to think about what they have experienced and learned, and importantly, why it was significant. Kegan [30], extends the concept of self-authoring, building on Mezirow’s (2000) theory of transformative learning which provides a theoretical basis on the role of the self. Such a pedagogical approach requires a learner to use critical reflection to “negotiate his or her own purposes, values, feelings and meanings…” ([31], p. 8).

It is evident from the perspectives of the health professional participants in this study that the *self*, based on concepts including personal attributes, respect and self-awareness, collectively enhances client-centred care and the individual’s capabilities for IPP. While educator participants recognised both the intraprofessional and interprofessional learning needs of students, they seemed to favour the *know* and *do* aspects (of IPE) and did not seem to emphasise the self as an important aspect of student development and transformation.

The framework developed by this research team initially focused on three primary interprofessional capabilities; specifically, client focus, working collaboratively and knowing one’s own and others’ roles [18]. These findings have highlighted the need for an added element in IPE curricula. As emphasised by practitioner participants, a focus on identification of, and reflection on, the concept of *self*, seems important. This could be considered as the intraprofessional dimension.

A focus on *self* in IPE may have a profound influence on how a student transitions from learning to *know and do*, to learning *to be* interprofessional. These findings may encourage educators and practitioners to move from thinking only about students learning about and demonstrating interprofessional capabilities, to developing a focus on facilitating students’ intraprofessional development. This additional focus might be of great value in preparing students in how *to be* an interprofessional practitioner.

The challenge for health professional educators seeking to promote the self in IPE is to understand how the self is impacted by students’ differences and idiosyncrasies, and how to enhance student self-reflection and self-awareness to develop their own awareness of self. Focussing on the *self* may encourage a greater understanding of the perceptions and values which underpin IPE and of how to facilitate student learning and preparation for interprofessional practice. This emphasis on learning to *be* an interprofessional practitioner may enable students to integrate the informal learning (the hidden curriculum) which occurs in the workplace as part of their development as a health professional.

Further research is needed to understand how students can learn about the self to become effective interprofessional practitioners, and how to support this learning across the range of different health professional disciplines, programs and contexts. This research would include developing a greater understanding of the elements involved in this transformation process, how it can be tailored to the range of student learning styles and dispositions, and how educators and practitioners can complement each other’s efforts in supporting students’ intraprofessional and interprofessional learning.

### Limitations of this study

Limitations of this study include participant self-selection. As described in the methods section, the action research approach used meant that new and previous participants were invited for each cycle, and so the same people did not necessarily take part in successive cycles. Also, although the findings of this study were verified through consensus amongst the research team, a limitation of this study is that participants were not further included as part of this subsequent analysis process. We have interpreted our findings through an educational focus. The findings could also be interpreted from other perspectives, such as from cultural differences. Some aspects of these factors (such as building a new cultural identity) have been addressed by this research group in an ethnographic study which looked at the complexity of integrating primary health services [32].

This study has not focussed on health professional students and future research could explore how students react and adapt to the differences identified in the discourse of educators and health professionals.

## Conclusion

The interprofessional language used by university educators and health practitioners in the field appears to be embedded in different values of practice. For the educators, the focus appears to be on what students know and do; whereas, for the health practitioners, the focus appears to be on who students are and what they bring to the interprofessional environment in their work with other practitioners and with clients.

These differences are evident in how each group represents interprofessional values and capabilities, how they position clients of health services within the learning and practice context, and the differing lens through which they perceive themselves and students. Educators from both academia and professional practice can assist students to bridge the differences between these two perspectives through a more formal emphasis on the *self* in the curriculum and through facilitating a transformation from *knowing about* interprofessional practice, to *being* the person who can become an effective practitioner within an interprofessional context.
